# The Impact of Open-Skill Exercises and E-Sports on Cognitive Function: A Narrative Review of Their Role in Preventing Cognitive Decline and Dementia

**DOI:** 10.3390/brainsci15070682

**Published:** 2025-06-25

**Authors:** Shuzo Kumagai, Hyuntae Park, Si Chen, Takao Yamasaki

**Affiliations:** 1 Kumagai Institute of Health Policy, Fukuoka 813-0044, Japan; 2 Institute of Digital Health Care, Dong-A University, Busan 49236, Republic of Korea; 3 Department of Health Care and Science, Dong-A University, Busan 49236, Republic of Korea; 4 Department of Nursing and Rehabilitation, Shandong University, Jinan 250012, China; 5 Department of Neurology, Minkodo Minohara Hospital, Fukuoka 811-2402, Japan

**Keywords:** cognitive dysfunction, dementia, cognition, exercise, physical activity, motor skills

## Abstract

**Background/Objectives:** There is still no clear consensus regarding the efficacy of exercise interventions in maintaining or improving cognitive function among independent older adults, as well as individuals with mild cognitive impairment (MCI) or dementia. This review explores the potential mechanisms underlying cognitive decline prevention and dementia mitigation from the perspective of motor learning theory, with a particular focus on aerobic-oriented open-skill exercise (OSE) and electronic sports (e-sports). **Methods:** Comprehensive literature searches were conducted using databases such as PubMed, Scopus, Web of Science, CiNii, and J-Stage (all available years) to identify studies examining the relationship between OSE, e-sports, and cognitive function. **Results:** Although various intervention studies have investigated aerobic exercise, resistance training, and other multifactorial exercise programs, a unified conclusion has not been reached regarding their effectiveness in enhancing cognitive function in the general elderly population or in patients with MCI or dementia. However, sports involving dynamic interaction with opponents (OSE) have shown a positive association with the maintenance and enhancement of cognitive abilities. Furthermore, e-sports present an accessible exercise modality, unrestricted by age, gender, time, or location, and are expected to support cognitive health in older adults. **Conclusions:** Aerobic-oriented OSE appears more effective than closed-skill exercise in preventing age-related cognitive decline and dementia. Additionally, e-sports may offer a comprehensive approach to brain health by integrating cognitive stimulation, physical engagement, social interaction, and stress reduction, though caution is advised regarding potential mental health concerns stemming from excessive use.

## 1. Introduction

Both domestically and internationally, the prevalence of mild cognitive impairment (MCI) and dementia is increasing alongside the aging population, not only in Europe and North America but also across various Asian countries [1]. In Japan, dementia ranks as the leading cause of conditions requiring long-term care certification [2]. Moreover, a meta-analysis has reported that the annual conversion rate from MCI diagnosed in medical institutions to all forms of dementia is no less than 9.6% [3]. Dementia not only negatively impacts the physical, psychological, social, and economic well-being of patients, but also places a significant burden on their families and caregivers. Furthermore, it greatly increases social costs, such as medical and nursing care expenses. Therefore, dementia-related care represents one of the most significant global challenges today [4]. These facts highlight the urgent need to prevent and manage MCI and dementia as critical public health issues.

Recent epidemiological studies in the field of physical activity research suggest that physical activity and exercise may help prevent cognitive decline and dementia [5,6,7,8,9]. However, there is still no clear consensus regarding the effectiveness of exercise interventions in independently living older adults or in individuals diagnosed with MCI or dementia. In addition, recent studies have examined the relationship between cognitive function and brain activity in the context of exercise and sports characteristics, irrespective of age or gender, from the perspective of motor skill demands [6,10]. This research has drawn considerable attention to the potential role of sports in preventing cognitive decline and dementia [6,10].

According to recent reviews, open-skill exercise (OSE; e.g., racket sports) is more effective than closed-skill exercise (CSE; e.g., running, swimming) in preventing and mitigating cognitive decline and dementia [10,11,12,13]. Furthermore, one study reported that individuals who participated in racket sports had a longer lifespan compared to those who were inactive or engaged in non-sport activities [14]. More recently, electronic sports (e-sports), including serious games [15], have been explored for their potential to enhance cognitive function and prevent cognitive decline, garnering increasing attention in this field [16]. Unlike traditional sports, e-sports can be played without restrictions on age, gender, time, or location, and they do not require physical contact or face-to-face interaction [17]. These unique characteristics suggest that e-sports participation may expand in the future.

Additionally, this review discusses the epidemiological study of motor skills, focusing on exposure variables that have not been previously analyzed. In this context, we provide an overview of motor skill classifications and summarize the characteristics of sports programs that are particularly suited for preventing cognitive impairment and dementia, including the potential benefits of e-sports. Finally, we explore the rationale behind recommending OSE for cognitive health, discussing its role in preventing and improving cognitive decline and its value—along with e-sports—as a lifelong physical activity.

The purpose of this narrative review is to summarize the effects of general exercise interventions for community-dwelling older adults, individuals with MCI, and those with dementia, and to present exercise guidelines appropriate for the prevention and improvement of cognitive function in these three groups. Furthermore, this review examines the effects of sports-related skill characteristics that may contribute to the maintenance and enhancement of cognitive function, as well as the potential role of e-sports in promoting cognitive health among older adults.

## 2. Methods: Literature Search Strategy

In Section 3, an electronic search was conducted for studies and information related to cognitive function using PubMed, Scopus, Web of Science, CiNii, and J-Stage (including all years). The search included a combination of the following keywords: “cognitive function”, “physical activity”, “exercise”, “dementia”, “MCI”, “elderly”, “exercise intervention”, and “guideline”. The titles and abstracts of the articles and the content of the websites were used to determine which articles were included and which were excluded. Articles and websites written in languages other than English were excluded, but an influential textbook written in Japanese was included [5,17]. In Section 4, the same literature search strategy was employed as in Section 3, but with the following additional keywords: “OSE”, “CSE”, and “therapy”. In Section 5, the same literature search strategy was also used, with the following new keywords: “e-sports”, “TV game”, “virtual reality”, “augmented reality”, and “artificial intelligence”.

In Section 3, we discussed the general effects of physical activity and exercise, excluding sports, on cognitive function in community-dwelling independent older adults and individuals with MCI and dementia, and summarized their mechanisms. Furthermore, we laid the groundwork for exploring specific types of exercise and sports in relation to cognitive function. In Section 4, we compared OSE and CSE to identify which types of exercise may be most beneficial for cognitive function and summarized their mechanisms. In Section 5, we provided a detailed description of e-sports, virtual games, and digital interventions for cognitive function. Furthermore, we extended the concept of OSE and e-sports-like experiences into modern and accessible formats.

## 3. Physical Activity Epidemiology for Dementia and Cognitive Decline

According to the 2024 report of the Lancet Commission on dementia prevention, intervention, and care [18], in addition to the previously identified 12 modifiable risk factors, vision loss and high low-density lipoprotein cholesterol have been added, bringing the total to 14 modifiable risk factors. Since physical inactivity is listed as a potential risk factor for dementia, it is recommended that adults aged 65 and older engage in 150 min of moderate-intensity physical activity or 75 min of vigorous-intensity physical activity per week [19]. In this section, we examine the effects of exercise interventions for independent older adults, as well as individuals with MCI and dementia, and introduce current physical activity and exercise guidelines for the prevention and management of MCI and dementia.

### 3.1. Effects of Exercise Interventions on Cognitive Function

A meta-analysis of randomized controlled trials (RCTs) in non-demented older adults found that aerobic exercise moderately improved cognitive functions, including attention, memory, and executive function [20]. However, findings remain inconsistent across studies. The LIFE Study [21] exemplifies this variability, showing no significant cognitive differences between long-term exercise interventions and health education programs after two years. These findings suggest that exercise interventions in late life may have limited effects on cognitive function, particularly in preventing the onset of MCI and dementia.

A systematic review of RCTs in individuals with MCI found that exercise interventions significantly improved language fluency, but no significant improvements were observed in other cognitive domains [22]. However, Suzuki et al. [23] designed a cognitive task-based exercise and conducted RCTs in Japanese MCI patients, showing improvements in processing speed and language ability. Furthermore, among individuals with amnestic MCI, exercise was reported to slow brain atrophy, suppress cognitive decline, and enhance memory.

A recent meta-analysis of RCTs on AD [24] suggested that physical activity and exercise can improve cognitive function or delay cognitive decline. However, methodological challenges remain, and the dose–response relationship between exercise intensity and cognitive improvement is still unclear. Some studies suggest that the total volume of daily physical activity may be more important than specific exercise routines for cognitive benefits [25], indicating a potential avenue for future research.

A Bayesian model-based network meta-analysis [26] examining the optimal exercise dosage and type for cognitive enhancement in older adults found a non-linear relationship between exercise volume and cognitive improvement. The study identified a minimum threshold of 700 METs min/week for clinically significant cognitive benefits. Additionally, resistance training required lower exercise volumes to achieve cognitive benefits compared to aerobic exercise, displaying an inverted U-shaped dose–response pattern.

The mechanism of cognitive function improvement associated with physical training is thought to be the improvement of physical fitness/motor function factors, nervous system factors, and circulatory system factors [27]. Improvement of physical fitness/motor function affects brain health through improvement of cardiovascular health and neurotrophic factors, leading to the maintenance of cognitive function. In particular, it has been reported that neurotrophic factors (brain-derived neurotrophic factor (BDNF) and Insulin growth factor-1), increased brain volume, neurogenesis, and synaptogenesis occur as nervous system factors, while cardiovascular factors such as increased capillaries (vascular endothelial growth factor), improved insulin resistance, reduced levels of inflammatory markers, and prevention of reduced cerebral blood flow contribute to cognitive function improvement [27].

Furthermore, Klimova and Dostalova [28] reviewed exercise interventions in healthy older adults, highlighting the unique cognitive benefits of dance-based exercise, which promotes neuroplasticity and reduces MCI progression risk. Notably, exercise interventions in MCI patients demonstrated improvements in executive function, delayed recall, and verbal fluency [29]. Mind–body exercises (e.g., Tai Chi, Baduanjin, and dance) were particularly effective. However, to date, no meta-analyses have examined the dose–response relationship between exercise and cognitive function in dementia populations.

Moreover, accumulating evidence across various clinical contexts suggests that dual-task interventions—integrating physical activity with simultaneous cognitive engagement—yield superior outcomes compared to either modality alone. These protocols have demonstrated particular efficacy in enhancing executive function, cognitive flexibility, and attentional control [30,31]. As such, dual-task strategies represent a promising non-pharmacological avenue for preserving and enhancing cognitive health in older adults, particularly those at risk for or experiencing MCI and AD. Nonetheless, further high-quality research is warranted to substantiate and refine these findings.

The evidence presented demonstrates general cognitive benefits of physical activity across populations. However, emerging research suggests that the type of motor skills involved in exercise may be equally important as the physiological adaptations. The following section examines how specific exercise characteristics—particularly the distinction between open-skill and closed-skill activities—influence cognitive outcomes through differential neural mechanisms.

### 3.2. Physical Activity and Exercise Guidelines for MCI and Dementia

Recently, a common international guideline on physical activity and exercise has been published to prevent and manage MCI and dementia [32]. Although physical activity and exercise have been proposed as effective interventions for preventing and managing MCI and dementia, no international guidelines have been established until now.

This guideline consists of the following three themes. Topic 1: Can physical activity and exercise delay the incidence of MCI or dementia in people without MCI or dementia? The guideline indicates that physical activity may contribute to dementia prevention, including AD and vascular dementia, but it is not necessarily more effective than an MCI education program in preventing cognitive decline. Although 100% of experts agree with this proposition, they acknowledge that the evidence remains uncertain. Therefore, physical activity should be considered as part of a multifactorial intervention. Experts also pointed out that physical activity and exercise interventions may complement other preventive measures.

Topic 2: Can physical activity and exercise delay the incidence of dementia in MCI patients? Currently, there is no conclusive evidence supporting the role of physical activity and exercise in slowing the progression from MCI to dementia. Additionally, 100% of experts agree that individuals with MCI who also have movement disorders should not be encouraged to engage in exercise.

Topic 3: Can physical activity and exercise improve cognition and impairment in dementia patients? The guideline suggests that patients with moderate dementia may consider physical activity and exercise as a means to maintain cognitive function. Compared to conventional care, exercise may help stabilize functional decline. Additionally, 86% of experts agree that physical activity and exercise are important for maintaining cognitive reserve and function in dementia patients. Furthermore, physical activity and exercise may have beneficial effects on emotional well-being, but these benefits should be balanced against potential risks.

The above summarizes the results of physical activity epidemiology research on preventing and mitigating cognitive decline and dementia. As pointed out in the preface, recent research from the perspective of sports skills has also accumulated, and Yamasaki [6] has reviewed this topic in a published article. Therefore, the research content and findings are introduced in the following sections.

## 4. Cognitive Function and Dementia Prevention, and Exercise and Sports Skills

### 4.1. Classification of Sports Skills

Sports skills can be broadly classified into OSE and CSE. While these categories provide a useful framework, many sports exhibit characteristics of both to varying degrees [10,33].

OSE refers to sports and physical activities performed in unpredictable environments that require adaptation to dynamic external rhythms. These sports, which fall under Categories 3 and 4 of exercise and sports characteristics (Table 1), demand synchronization with external rhythms and pace, greater cognitive and decision-making demands, and continuous adaptive movement. Representative OSE sports include table tennis, tennis, and badminton [10].

In contrast, CSE refers to exercises performed in relatively stable, self-regulated, and predictable environments. These activities, classified into Categories 1 and 2 (Table 1), include swimming, running, and cycling. The primary characteristics of CSE involve self-paced rhythms, minimal cognitive demands related to external stimuli, and fewer decision-making elements [10].

Rather than forming distinct divisions, OSE and CSE exist on a continuum, representing two ends of a spectrum. Higher category numbers (e.g., Category 4) indicate a greater emphasis on open skills, whereas lower category numbers (e.g., Category 1) indicate a greater emphasis on closed skills.

### 4.2. Differential Effects of OSE and CSE on Cognitive Function

Recent review papers have suggested that, compared to CSE, OSE is not only the most effective exercise type for maintaining cognitive function in healthy, independent older adults but also beneficial for younger individuals in preventing cognitive decline and stimulating brain activity [10,33]. In addition, the Copenhagen City Heart Study reported that OSE is associated with increased lifespan [14]. Whether this association extends to cognitive decline prevention and dementia risk reduction remains an interesting research question.

OSE and CSE differ significantly in terms of environmental stability and cognitive demands [10]. This distinction has been explored in recent studies [11,12,33]. In a systematic review by Gu et al. [11], 19 studies (14 observational studies and 5 intervention studies; participants aged 9.6 to 70.5 years) were analyzed. Among the observational studies, 7 out of 14 reported superior cognitive benefits of OSE compared to CSE. In the intervention studies, three out of five demonstrated that OSE improved cognitive functions such as visuospatial attention, problem-solving, auditory perception, inhibitory control, and cognitive flexibility in both children and older adults.

A meta-analysis by Zhu et al. [12] reviewed 19 studies (15 cross-sectional studies and 4 intervention studies; participants aged 9.6 to 69.4 years). The cross-sectional studies found that OSE had a greater effect on cognitive functions, particularly inhibitory control and cognitive flexibility, compared to CSE. However, intervention studies showed no significant difference between OSE and CSE in improving cognitive function, highlighting the need for long-term studies to confirm OSE’s effectiveness.

Another meta-analysis and systematic review by Heilmann [33], which included 19 studies (participants aged 10.2 to 69.9 years), confirmed that OSE was more effective than CSE in enhancing executive functions, with cognitive flexibility showing the greatest improvement, followed by inhibitory control and working memory. These findings demonstrate that OSE produces beneficial effects on cognitive function across all age groups, with consistent benefits observed in older adults [11,12,33].

### 4.3. The Mechanism of Cognitive Function Improvement by OSE

This section describes the mechanisms underlying cognitive function improvement. There are many different sports in the OSE (Table 1), but in this section, we introduce key examples such as racket sports (e.g., table tennis and badminton) [10,34] and martial arts (e.g., judo) [35].

Aerobic exercise is generally known to improve cardiovascular risk factors, enhance neurotrophic factor expression, promote amyloid beta metabolism, increase cerebral blood flow, and reduce inflammation, all of which contribute to maintaining and improving cognitive function [10].

Among racket sports, table tennis is characterized by moderate-intensity aerobic activity (approximately 96%). It has been shown to induce neuroplastic changes in multiple brain networks, including motor-related and visual processing regions (particularly the motion-visual areas), as well as the frontal cortex. These neuroplastic adaptations help maintain or improve cognitive functions, particularly executive function and sensorimotor coordination, thereby reducing the risk of age-related cognitive decline and dementia [10]. Badminton, which relies primarily on aerobic metabolism (60–70%) [34], has been associated with increased cerebellar gray matter volume and functional changes in the frontoparietal junction, which may enhance visuospatial integration in players.

Judo, classified as an aerobic sport (approximately 70%), also induces neuroplasticity through similar mechanisms to racket sports. Judo training has been shown to increase gray matter volume in key brain areas, including the frontal lobe (responsible for planning and executing movement), prefrontal cortex (involved in working memory and cognitive processing), parietal and occipital lobes (which process visual information), and the middle and inferior temporal gyri (associated with motor learning and memory) [35]. Additionally, judo training enhances functional connectivity in key neural networks, such as the sensorimotor network (involved in movement preparation and execution), visual network (critical for target positioning and motion trajectory tracking), and cerebellar network (which regulates posture, balance, and movement coordination) [35]. These neural adaptations contribute to improved cognitive functions, such as memory and executive function, as well as physical performance, including fall prevention [35].

These findings demonstrate that aerobic OSE provides superior cognitive protection compared to CSE by enhancing neural network activation and promoting neuroplasticity mechanisms [10,33]. The superior effectiveness of OSE can be attributed to three complementary advantages over traditional CSE: (1) enhanced cognitive engagement through real-time decision-making and adaptive problem-solving, (2) sustained motivational engagement due to unpredictable and dynamic environments, and (3) simultaneous activation of multiple cognitive domains, including executive function, working memory, and attention control.

Among OSE options, table tennis emerges as particularly promising due to its optimal combination of cognitive demands, accessibility, and safety profile [10]. However, the wide variety of OSE activities (Table 1) allows for personalized intervention selection based on individual preferences and abilities. Importantly, emerging evidence supports incorporating OSE into lifestyle-based approaches that integrate multiple health-promoting behaviors. While further research is needed to determine optimal combinations, the multifactorial nature of cognitive decline suggests that multimodal interventions may provide enhanced neuroprotective benefits [10]. Having established the cognitive superiority of OSE through traditional sports modalities, we now turn to exploring how e-sports—as digital extensions of open-skill-like cognitive demands—may offer similar neuroprotective benefits with improved accessibility and engagement.

## 5. Cognitive Function and E-Sports

The global rise in age-related cognitive impairments has necessitated innovative approaches to maintaining and enhancing cognitive health. MCI and dementia pose significant public health challenges with profound economic and social implications, particularly in rapidly aging societies such as Japan and South Korea [36]. This issue is further complicated by the bidirectional relationship between cognitive impairment and frailty in older adults. Park et al. [37] demonstrated significant associations between physical function, mental function, and frailty status in community-dwelling older adults, highlighting the complex interplay between these factors.

While conventional exercise interventions have been shown to enhance cognitive function, they face significant limitations in terms of long-term sustainability and participant adherence. Di Lorito et al. [38] conducted a systematic review and meta-analysis, revealing that adherence to exercise interventions among older adults with MCI and dementia often declines substantially over time, with dropout rates reaching 17–18%. This decline is largely attributed to motivational barriers. O’Neil-Pirozzi et al. [39] emphasized that motivation is a critical determinant of both initiating and adhering to physical and cognitive exercise programs in older adults. Traditional interventions often fail to sustain engagement due to perceived monotony, lack of immediate feedback, and insufficient psychosocial reinforcement. These factors can significantly undermine the effectiveness of programs, regardless of their theoretical benefits.

In response to these challenges, e-sports integrated with advanced technologies have emerged as a promising approach to cognitive rehabilitation and enhancement, offering potential solutions to the motivational and adherence limitations of conventional interventions.

E-sports, defined as competitive video gaming conducted in structured environments, go beyond traditional gaming by incorporating organized competition and sophisticated technologies such as virtual reality (VR), augmented reality (AR), and artificial intelligence (AI). These technologies transform gaming into cognitively demanding experiences. Unlike traditional exercise interventions, which often struggle with participant retention and engagement [38], e-sports leverage intrinsic motivational elements—including competition, achievement, social interaction, and immersion—that can significantly enhance adherence and long-term participation. This strong motivational foundation addresses a critical gap in conventional cognitive interventions, potentially leading to improved long-term outcomes through sustained engagement [39].

This section examines the growing body of evidence supporting e-sports as effective tools for enhancing cognitive function while identifying current research gaps and future directions in this rapidly evolving field.

### 5.1. The Neurocognitive Demands of E-Sports

E-sports engage multiple cognitive domains simultaneously, creating complex neural activation patterns that promote brain health. Research by Campbell et al. [40] highlights e-sports as a unique window into neurocognitive expertise, demonstrating that professional gamers exhibit enhanced visual attention, cognitive flexibility, and information processing capabilities compared to non-gamers. The cognitive architecture recruited during e-sports participation extends beyond simple reaction time and encompasses higher-order cognitive processes such as strategic planning, decision-making under time constraints, and adaptive problem-solving.

When combined with technologies such as VR and AR, e-sports create immersive environments that intensify cognitive engagement. Lachowicz et al. [41] documented significant improvements in concentration and alternating attention following short-term VR training among e-athletes. These findings suggest that technological enhancements in gaming environments may amplify the cognitive benefits derived from e-sports participation. The sensory-rich environments created by these technologies stimulate multiple neural pathways, facilitating cross-modal integration and cognitive flexibility.

### 5.2. Neurobiological Mechanisms Underlying Cognitive Enhancement

Building on the established cognitive benefits of OSE, engagement in e-sports activates similar, yet technologically enhanced, neurobiological pathways. The cognitive benefits associated with e-sports participation can be attributed to several key neurobiological mechanisms. Foremost among these is neuroplasticity—the brain’s ability to reorganize itself by forming new neural connections. E-sports, particularly those incorporating VR technology, have been shown to elicit enhanced neuroplastic responses compared to conventional cognitive training, as evidenced by structural and functional adaptations in brain regions associated with attention, memory, and executive function [42]. These amplified structural and functional adaptations provide the neural foundation for cognitive enhancement.

Beyond neuroplasticity, e-sports participation enhances cerebral blood flow, ensuring optimal delivery of oxygen and nutrients to neural tissues. This increased perfusion supports the metabolic demands of active neural networks and promotes the release of neurotrophic factors such as BDNF. BDNF plays a crucial role in learning, memory formation, and neuroprotection, acting as a molecular mediator of experience-dependent plasticity. The immersive nature of VR-enhanced e-sports may further augment these effects by creating emotionally engaging experiences that modulate arousal and attention networks [43].

Additionally, the stress-reducing effects of e-sports contribute to cognitive enhancement by regulating the hypothalamic–pituitary–adrenal axis. Chronic stress is associated with hippocampal atrophy and impaired cognitive performance, particularly in memory-related domains. The engaging and rewarding nature of e-sports can mitigate stress responses, thereby preserving cognitive function through neuroendocrine pathways. This stress regulation mechanism may be particularly relevant in light of Bae et al.’s [44] findings on the trajectories of subjective cognitive decline and frailty in older adults, where psychological factors were found to significantly influence cognitive health trajectories.

### 5.3. Domain-Specific Cognitive Effects of E-Sports

Executive function encompasses cognitive processes essential for goal-directed behavior, including working memory, inhibitory control, and cognitive flexibility. Research by Sañudo et al. [45] demonstrated that aerobic exercise combined with VR significantly improved cognitive flexibility and selective attention in young males. This finding suggests that integrating physical activity with virtual environments—a growing trend in e-sports—may particularly enhance executive control networks.

Furthermore, Toth et al. [46] provided converging evidence for the cognitive link between exercise and e-sports performance. Their dual systematic review revealed that executive functions trained through e-sports transfer to physical performance contexts and vice versa, suggesting shared neural substrates. This bidirectional relationship highlights the potential for synergistic effects when e-sports are combined with physical activity interventions. Such synergistic effects are further supported by Thapa et al. [47], who demonstrated that a combination of electrical muscle stimulation and resistance exercise significantly improved both physical and cognitive function in middle-aged and older women, suggesting multiple pathways through which physical activation enhances brain function.

Memory systems—including episodic, semantic, and procedural memory—also benefit significantly from e-sports engagement. VR-based cognitive interventions have shown promise in enhancing memory retention and spatial navigation abilities. A meta-analysis by Rosa et al. [48] examining the effects of VR-based serious games in cognitive interventions found moderate to large effect sizes for memory improvement, particularly in older adults and individuals with cognitive impairments.

The immersive nature of VR environments facilitates context-dependent learning and memory encoding. By creating rich associative networks, these environments enhance both the acquisition and retrieval of information. Thapa et al. [49] demonstrated that a VR-based intervention program significantly improved cognition in older adults with MCI, with particular benefits for visuospatial memory and episodic recall. These memory enhancements may be especially valuable given Bae et al.’s [44] observation that subjective cognitive decline is an early indicator of potential frailty development in community-dwelling older adults, suggesting that memory-focused interventions could serve as preventative measures against broader functional decline.

Attentional networks—including sustained, selective, and divided attention—are primary targets for e-sports-based cognitive enhancement. Lachowicz et al. [41] found that short-term VR training significantly improved concentration and alternating attention in e-athletes. These improvements in attentional control translate to enhanced information processing capabilities across multiple cognitive domains.

Processing speed, a fundamental cognitive resource that underpins higher-order cognition, also benefits from e-sports engagement. Stanmore et al. [50] conducted a meta-analysis examining the effects of active video games on cognitive function and found significant improvements in processing speed across diverse populations. These effects may be particularly beneficial for older adults, for whom reduced processing speed often represents an early marker of cognitive decline. Park et al. [37] highlighted the interrelationship between cognitive processing speed and physical function, noting that interventions targeting either domain often yield benefits in both. This finding suggests that integrated approaches may provide optimal outcomes for preserving functional independence.

### 5.4. E-Sports Applications for the Elderly

E-sports can be applied across all stages of life, but this section focuses on their applications for older adults. Perhaps the most compelling benefits of e-sports for cognitive enhancement lie in aging populations and individuals with cognitive impairments. Yang et al. [36] demonstrated that VR and exercise training improved brain function, cognitive abilities, and physical health in older adults with MCI. Their RCTs found improvements across multiple cognitive domains, suggesting potential for both the prevention and remediation of age-related cognitive decline.

Ferreira et al. [51] further confirmed the efficacy of multimodal exercise combined with AR for cognitive enhancement in community-dwelling older adults. Their findings highlight the potential for technology-enhanced interventions to support cognitive health in naturalistic settings, potentially expanding access to cognitive rehabilitation resources. This approach aligns with Park et al.’s [37] findings on the interrelationship between physical function, cognitive function, and frailty, suggesting that integrated interventions addressing multiple domains simultaneously may yield optimal outcomes for preserving independence in older adults.

For clinical populations with neurodegenerative conditions, e-sports offer promising avenues for cognitive rehabilitation. Sokolov et al. [52] reviewed serious video games and VR applications for preventing and treating cognitive decline due to aging and neurodegeneration. Their work suggests that well-designed gaming interventions may complement traditional therapeutic approaches, potentially improving outcomes through increased engagement and adherence. Given Bae et al.’s [44] observation that subjective cognitive decline and frailty trajectories are influenced by modifiable factors, e-sports interventions targeting these pathways may serve as valuable tools for altering unfavorable cognitive aging trajectories.

### 5.5. Technological Innovations Enhancing Cognitive Impact

#### 5.5.1. Integration

The evolution of VR technology has significantly expanded the cognitive potential of e-sports. Sakhare et al. [53] demonstrated that combining physical exercise with cognitive training in VR positively impacts brain health and cognition in older adults. This integration of physical and cognitive demands within immersive environments represents a major advancement over traditional cognitive training approaches. The neurobiological mechanisms underlying the effectiveness of VR include enhanced sensory integration, embodied cognition, and presence—the subjective experience of being in a virtual environment. These factors collectively intensify cognitive engagement, potentially accelerating neural adaptations and skill acquisition. Richlan et al. [54] reviewed the efficacy of virtual training for sports performance enhancement and found that VR-based interventions produced “real effects”, suggesting a strong transfer of skills from virtual to real-world contexts.

#### 5.5.2. Applications

AR technology overlays digital information onto the physical world, creating hybrid environments that simultaneously engage both physical and cognitive systems. Ferreira et al. [51] demonstrated that multimodal exercise combined with AR significantly improved cognition in community-dwelling older adults. The spatial mapping required to interact with AR environments may be particularly beneficial for visuospatial processing and spatial cognition—cognitive domains often affected in the early stages of dementia.

The accessibility of AR applications, which often require only a smartphone or tablet rather than specialized hardware, may facilitate broader implementation of cognitive enhancement programs. This accessibility, combined with the potential for integrating AR elements into daily activities, positions AR-enhanced e-sports as promising tools for reinforcing “everyday cognition”. This aligns with Park et al.’s [37] emphasis on the importance of implementing interventions that can be seamlessly incorporated into community settings to maximize their reach and public health impact.

#### 5.5.3. Adaptive Algorithms and Personalization

AI integration represents the next frontier in e-sports-based cognitive enhancement. Adaptive algorithms capable of adjusting difficulty levels in real time optimize the challenge-skill balance—a critical factor in maintaining cognitive engagement and achieving flow states. These personalized approaches ensure that participants consistently operate at the edge of their capabilities, maximizing cognitive demand without inducing frustration or disengagement.

Additionally, AI-driven systems can track performance metrics across various cognitive domains, identifying specific areas for targeted intervention. This precision-based approach to cognitive enhancement represents a significant improvement over one-size-fits-all cognitive training programs, potentially increasing both effectiveness and efficiency. The potential for personalization is particularly relevant given Bae et al.’s [44] observation that individual trajectories of cognitive decline and frailty vary substantially. This finding suggests the need for tailored interventions that address specific risk factors and functional profiles.

### 5.6. Cognitive Benefits of Different E-Sports Game Genres

The exponential growth of e-sports has generated substantial scholarly interest in understanding the cognitive implications of video game engagement. Contemporary research has transcended simplistic evaluations of gaming as a unitary activity, moving toward a more nuanced examination of genre-specific cognitive effects. Empirical evidence increasingly suggests that various e-sports game genres enhance cognitive abilities in distinctive and specialized ways, offering potential applications for targeted cognitive training interventions.

Recent investigations indicate that First-person Shooter (FPS) games significantly enhance reaction time and selective attention, improving visual information processing capabilities and working memory capacity [55,56]. The immersive perceptual demands of FPS games train attentional control mechanisms, resulting in measurable improvements in visuospatial processing. Additionally, FPS gaming demonstrates positive effects on spatial reasoning and mental rotation abilities [57].

Multiplayer Online Battle Arena (MOBA) games effectively enhance strategic decision-making abilities and cognitive flexibility, particularly complex working memory and sustained attention necessary for team-based strategy execution [57,58]. Elite MOBA gamers demonstrate superior abilities in maintaining continuous attention and handling multiple tasks simultaneously during gameplay [59]. The coordination of individual actions within team strategies appears to enhance both cognitive and social information processing pathways.

Real-time Strategy (RTS) games show excellent effects on improving planning abilities, cognitive flexibility, and multitasking capabilities. These games positively influence strategic thinking and problem-solving skills through their requirements for resource management and tactical adaptation [60]. RTS gamers exhibit superior complex decision-making abilities required for general spatial reasoning and long-term strategic planning [61].

Sports simulation games effectively enhance real-time response capabilities, sustained attention, and spatial cognition, while also contributing to the development of pattern recognition and strategic memory [62,63]. These games improve players’ psychomotor abilities and strategic judgment skills in ways that parallel actual sports participation [64].

VR games have recently gained scholarly attention as effective mechanisms for cognitive enhancement. VR-based exercise and cognitive training for elderly individuals with MCI positively impact cognitive function, physical health, and brain activation [36,49]. The embodied cognition facilitated by VR environments may offer unique advantages for cognitive training, particularly for populations with existing cognitive vulnerabilities.

The emerging consensus suggests that different cognitive abilities are emphasized according to game genre, highlighting the importance of selecting appropriate games for specific objectives when designing game-based cognitive training programs [65]. This genre-specific approach to understanding cognitive benefits offers promising avenues for targeted interventions across developmental, educational, and clinical domains.

### 5.7. Negative Consequences of Excessive E-Sports Engagement

Despite the documented cognitive benefits of strategic gaming, excessive e-sports engagement presents significant biopsychosocial concerns. Research indicates that problematic gaming behavior correlates with impaired academic and occupational performance [66], with competitive gaming potentially displacing educational and developmental activities during formative years. Sleep architecture disruption constitutes another critical issue, as extended gaming sessions and screen exposure suppress melatonin production, compromising both sleep quality and quantity [67], which subsequently impairs cognitive functioning and emotional regulation.

Paradoxically, while moderate gaming may enhance specific cognitive domains, excessive engagement appears to impair certain cognitive functions. Research by Moisala et al. [68] demonstrates that excessive media multitasking, common in intensive gaming environments, is associated with increased distractibility and diminished attentional control. Sustained attention to e-sports can lead to attentional bias, where cognitive resources become disproportionately allocated to gaming-related stimuli at the expense of other environmental inputs. Furthermore, Takeuchi et al. [69] found that excessive screen time correlates with reduced prefrontal cortex development in adolescents, potentially affecting executive functions including working memory, cognitive flexibility, and inhibitory control. The dopaminergic reward system activation during gaming, when overstimulated, may result in neuroadaptations that elevate reward thresholds, potentially diminishing motivation for less immediately rewarding cognitive tasks [70].

The sedentary nature of intensive gaming contributes to physical health complications, including increased metabolic syndrome risk factors and musculoskeletal disorders, particularly repetitive strain injuries affecting the upper extremities [71]. Psychologically, meta-analytic evidence reveals significant associations between problematic gaming and mood disorders, including depression and anxiety [72], with bidirectional causal pathways hypothesized. Social functioning may deteriorate despite the superficially social nature of multiplayer gaming, as virtual interactions inadequately substitute for face-to-face social development, potentially exacerbating loneliness and social isolation [73].

The World Health Organization’s recognition of gaming disorder in the ICD-11 as a mental health condition, characterized by impaired control and continued gaming despite negative consequences, underscores the clinical significance of these concerns. Importantly, these adverse outcomes manifest primarily in excessive, obsessive engagement rather than moderate participation, following a curvilinear relationship where moderate gaming may confer benefits while excessive involvement yields diminishing returns [74]. These findings emphasize the necessity of balanced approaches to e-sports participation, incorporating appropriate limitations and complementary activities to mitigate potential harms while preserving cognitive benefits.

### 5.8. Research Gaps and Future Directions

Despite promising evidence supporting e-sports as tools for cognitive enhancement, several research gaps remain. Longitudinal studies examining sustained cognitive benefits are scarce, limiting our understanding of long-term outcomes. The optimal parameters for e-sports interventions—including intensity, duration, frequency, and specific game characteristics—are still not well defined.

The current literature demonstrates substantial heterogeneity in study design, which significantly affects the quality of evidence and its clinical applicability. Intervention studies and observational studies present fundamental methodological differences: observational studies provide evidence of associations, while intervention studies offer causal inferences but often suffer from insufficient sample sizes and short follow-up durations. This inconsistency in study design hinders robust effect size estimation and the development of clinical guidelines, as meta-analyses are impeded by methodological disparities between studies. A critical limitation of current research is the lack of long-term outcome assessments. The paucity of studies with follow-up periods exceeding five years severely restricts our understanding of sustained cognitive protection. Existing evidence primarily demonstrates short-term cognitive improvements (over weeks to months); however, whether OSE and e-sports confer lasting neuroprotection against age-related cognitive decline or dementia onset remains unclear. Another major research need is the standardization of outcome measures for effective comparison. Significant heterogeneity in cognitive assessment tools—ranging from brief screening instruments (e.g., Mini-Mental State Examination, Montreal Cognitive Assessment) to comprehensive neuropsychological batteries—fundamentally undermines cross-study comparison and meta-analytic synthesis. The development of standardized and validated cognitive assessment protocols specifically tailored to OSE and e-sports research is urgently required to facilitate meaningful comparisons of effect sizes and translation into clinical practice. Additionally, individual difference factors that may moderate intervention effectiveness require further investigation. Age, baseline cognitive status, technology familiarity, and genetic factors could all influence responsiveness to e-sports-based cognitive interventions. Understanding these moderating variables will enable more precise targeting of interventions to those most likely to benefit. Bae et al. [44] identified several factors influencing the trajectories of subjective cognitive decline and frailty in older adults, including psychological variables and physical activity levels. Future research should explore how these factors might interact with e-sports interventions to determine which subpopulations would derive the greatest benefit.

Standardization of outcome measures is another critical research need. The heterogeneity of cognitive assessments across studies complicates cross-study comparisons and meta-analyses. Developing standardized cognitive assessment batteries specifically for e-sports research would facilitate more rigorous evaluation of intervention efficacy. Park et al. [37] emphasized the importance of comprehensive assessment approaches that capture both cognitive and physical function domains. Future e-sports research should adopt similar multi-domain assessment protocols to fully characterize intervention effects.

Additionally, research should explore the potential synergies between e-sports and other cognitive enhancement strategies. Given the emerging evidence from multimodal intervention studies [68], integrating e-sports with complementary lifestyle factors may optimize cognitive outcomes through additive or synergistic mechanisms. Sleep optimization represents a particularly relevant complementary intervention, as e-sports engagement patterns can influence circadian rhythms and sleep architecture. Poor sleep quality may attenuate the cognitive benefits of both physical and cognitive training, whereas adequate sleep facilitates memory consolidation and neuroplasticity—key mechanisms underlying cognitive improvement associated with OSE and e-sports participation. Nutritional strategies also warrant consideration as adjunctive approaches. Mediterranean diet patterns and omega-3 supplementation have demonstrated neuroprotective effects that may enhance the cognitive benefits derived from OSE and e-sports by improving BDNF expression and reducing neuroinflammation. For specific clinical populations, evidence-based pharmacological interventions or medical management may complement lifestyle-based approaches, although careful investigation of potential interaction effects remains essential [75]. Multimodal approaches may yield greater cognitive benefits than any single intervention alone by simultaneously targeting multiple mechanisms involved in cognitive decline. Thapa et al. [47] demonstrated enhanced outcomes through a combination of electrical muscle stimulation and resistance exercise, suggesting that integrating e-sports with other modalities may produce synergistic benefits.

While traditional exercise-based interventions have well-documented cognitive benefits, their effectiveness is often limited by adherence challenges and motivational barriers [38,39]. E-sports and technology-enhanced interventions address these limitations through their inherently engaging, customizable, and socially interactive qualities. The immersive and gamified nature of e-sports provides immediate feedback, progressive challenge calibration, and intrinsic reward structures, all of which significantly enhance motivation and long-term adherence—critical factors that conventional approaches often fail to adequately address.

As technology continues to evolve and research progresses in addressing these gaps, e-sports may increasingly become a central component of comprehensive cognitive health strategies. Their ability to combine cognitive engagement with physical activity, social interaction, and stress reduction positions them as a uniquely holistic approach to brain health. Given the established interrelationships between physical function, cognitive function, and frailty status [31], as well as the observed influence of modifiable factors on cognitive and functional trajectories [44], e-sports represent a versatile intervention modality capable of addressing multiple dimensions of health simultaneously.

## 6. Limitations

This narrative review has several limitations that warrant consideration. First, as a narrative review, this study may have been subject to subjective bias in the process of literature selection and analysis. Although we sought to cover key studies, a systematic search strategy was not employed, which may have led to the omission of relevant literature.

Second, due to limitations in the types of studies included, we did not perform a formal quality assessment or conduct a meta-analysis, and therefore could not quantify the consistency of results or estimate effect sizes.

Third, significant heterogeneity across studies substantially limits the generalizability of findings. This heterogeneity encompasses participant characteristics (e.g., age, MCI subtype), intervention design (e.g., exercise type, intensity, duration), and cognitive assessment tools. For example, discrepancies in outcome measures (e.g., varying neuropsychological tests) and protocol variability (e.g., 30 min vs. 90 min sessions) hinder cross-study comparability, as highlighted in meta-analyses noting unclear dose–response patterns.

Fourth, some of the original studies were observational in nature and could not rule out the influence of confounding factors (e.g., genetic background, socioeconomic status) on the association between exercise and cognition. Therefore, the conclusions indicate association rather than causation. Additionally, the differential effect sizes between intervention and observational studies may reflect these methodological differences in controlling for potential confounders.

Fifth, the lack of standardized cognitive assessment protocols across studies severely hampers cross-study comparisons and meta-analytic efforts. Different studies employ varying neuropsychological test batteries, assessment timing, and outcome definitions, preventing the establishment of evidence-based dosage recommendations for both OSE and e-sports applications.

Sixth, long-term follow-up data (>5 years) are critically scarce, limiting insights into sustained cognitive protection and dementia prevention. Most intervention studies examine cognitive effects over 6 to 24 months, providing insufficient evidence for establishing long-term preventive benefits. This temporal limitation is particularly problematic given that cognitive decline typically occurs over decades.

Lastly, clinical populations remain underrepresented, with samples biased toward healthy older adults or individuals with early-stage MCI. There is a lack of representation from individuals with advanced MCI, early-onset dementia, and from diverse geographic and socioeconomic backgrounds. Future research should address these limitations through systematic reviews, meta-analyses with standardized outcome measures, high-quality RCTs with longer follow-up periods, and studies involving underrepresented populations to strengthen causal evidence and enhance generalizability.

## 7. Conclusions

This review first summarizes research findings in physical activity epidemiology from the perspective of preventing and reducing the incidence of MCI and dementia. In particular, it explores the potential mechanisms underlying cognitive decline prevention and dementia mitigation through motor learning theory, focusing on aerobic-oriented OSE and e-sports.

To date, epidemiological studies have primarily investigated the effects of physical activity and exercise in terms of energy metabolism and physiological adaptations. However, this review highlights the cognitive significance of OSE and e-sports within the context of aerobic exercise. Looking ahead, it remains an important research question whether increased participation in OSE and e-sports across all age groups could help prevent cognitive decline and reduce the incidence of dementia, or conversely, whether such participation could enhance neuroplasticity in younger populations.

Furthermore, there is a pressing need to develop innovative age-inclusive participation frameworks designed to foster intergenerational competitive engagement. Addressing this challenge will be instrumental in optimizing the enduring benefits that OSE and e-sports may offer for maintaining cognitive health across the lifespan.

## Figures and Tables

**Table 1:** Classification of sports based on skill type.

Skill Type and Its Continuum	Category	Sports
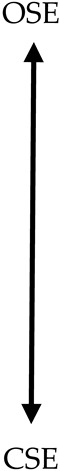	4	Tennis, Table Tennis, Badminton, Basketball, Volleyball, Soccer, Handball, American Football, Wushu, Martial Arts, Fencing, Korfball, Hockey, Baseball
	3	Sailing, Canoe Slalom
	2	Athletics, Cross-country Skiing
	1	Swimming, Running, Triathlon, Cycling, Gymnastics, Archery, Shooting, Brisk Walking, Track Bike

Abbreviation: CSE, closed-skill exercise; OSE, open-skill exercise.

## Data Availability

No new data were created or analyzed in this study.

## References

[1] Tahami Monfared A.A., Byrnes M.J., White L.A., Zhang Q. (2022). Alzheimer’s Disease: Epidemiology and Clinical Progression. Neurol. Ther..

[2] Comprehensive Survey of Living Conditions. https://www.mhlw.go.jp/english/database/db-hss/cslc-index.html.

[3] Mitchell A.J., Shiri-Feshki M. (2009). Rate of Progression of Mild Cognitive Impairment to Dementia--Meta-Analysis of 41 Robust Inception Cohort Studies. Acta Psychiatr. Scand..

[4] World Health Organization Dementia. https://www.who.int/news-room/fact-sheets/detail/dementia.

[5] Shimada H., Hotta R. (2016). Physical Activity Epidemiology in Nursing Care Service: Dementia. Physical Activity and Sedentary Behavior Science.

[6] Hernández-Mendo A., Reigal R.E., López-Walle J.M., Serpa S., Samdal O., Morales-Sánchez V., Juárez-Ruiz de Mier R., Tristán-Rodríguez J.L., Rosado A.F., Falco C. (2019). Physical Activity, Sports Practice, and Cognitive Functioning: The Current Research Status. Front. Psychol..

[7] Srinivas N.S., Vimalan V., Padmanabhan P., Gulyás B. (2021). An Overview on Cognitive Function Enhancement through Physical Exercises. Brain Sci..

[8] Iso-Markku P., Kujala U.M., Knittle K., Polet J., Vuoksimaa E., Waller K. (2022). Physical Activity as a Protective Factor for Dementia and Alzheimer’s Disease: Systematic Review, Meta-Analysis and Quality Assessment of Cohort and Case–Control Studies. Br. J. Sports Med..

[9] Tari A.R., Walker T.L., Huuha A.M., Sando S.B., Wisloff U. (2025). Neuroprotective Mechanisms of Exercise and the Importance of Fitness for Healthy Brain Ageing. Lancet.

[10] Yamasaki T. (2023). Preventive Strategies for Cognitive Decline and Dementia: Benefits of Aerobic Physical Activity, Especially Open-Skill Exercise. Brain Sci..

[11] Gu Q., Zou L., Loprinzi P.D., Quan M., Huang T. (2019). Effects of Open Versus Closed Skill Exercise on Cognitive Function: A Systematic Review. Front. Psychol..

[12] Zhu H., Chen A., Guo W., Zhu F., Wang B. (2020). Which Type of Exercise Is More Beneficial for Cognitive Function? A Meta-Analysis of the Effects of Open-Skill Exercise versus Closed-Skill Exercise among Children, Adults, and Elderly Populations. Appl. Sci..

[13] Guo W., Wang B., Smoter M., Yan J. (2021). Effects of Open-Skill Exercises on Cognition on Community Dwelling Older Adults: Protocol of a Randomized Controlled Trial. Brain Sci..

[14] Schnohr P., O’Keefe J.H., Holtermann A., Lavie C.J., Lange P., Jensen G.B., Marott J.L. (2018). Various Leisure-Time Physical Activities Associated with Widely Divergent Life Expectancies: The Copenhagen City Heart Study. Mayo Clin. Proc..

[15] Saragih I.D., Everard G., Lee B.-O. (2022). A Systematic Review and Meta-Analysis of Randomized Controlled Trials on the Effect of Serious Games on People with Dementia. Ageing Res. Rev..

[16] Stern Y., Blumen H.M., Rich L.W., Richards A., Herzberg G., Gopher D. (2011). Space Fortress Game Training and Executive Control in Older Adults: A Pilot Intervention. Neuropsychol. Dev. Cogn. B Aging Neuropsychol. Cogn..

[17] Mizukuni T. (2022). A Study on the Effectiveness of E-Sports as Cognitive Training for the Elderly and Their Motivation to Participate. J. Int. ICT Appl. Res. Soc..

[18] Livingston G., Huntley J., Liu K.Y., Costafreda S.G., Selbæk G., Alladi S., Ames D., Banerjee S., Burns A., Brayne C. (2024). Dementia Prevention, Intervention, and Care: 2024 Report of the Lancet Standing Commission. Lancet.

[19] WHO Guidelines on Physical Activity and Sedentary Behaviour: At a Glance. https://www.who.int/europe/publications/i/item/9789240014886.

[20] Smith P.J., Blumenthal J.A., Hoffman B.M., Cooper H., Strauman T.A., Welsh-Bohmer K., Browndyke J.N., Sherwood A. (2010). Aerobic Exercise and Neurocognitive Performance: A Meta-Analytic Review of Randomized Controlled Trials. Psychosom. Med..

[21] Sink K.M., Espeland M.A., Castro C.M., Church T., Cohen R., Dodson J.A., Guralnik J., Hendrie H.C., Jennings J., Katula J. (2015). Effect of a 24-Month Physical Activity Intervention vs Health Education on Cognitive Outcomes in Sedentary Older Adults: The LIFE Randomized Trial. JAMA.

[22] Gates N., Fiatarone Singh M.A., Sachdev P.S., Valenzuela M. (2013). The Effect of Exercise Training on Cognitive Function in Older Adults with Mild Cognitive Impairment: A Meta-Analysis of Randomized Controlled Trials. Am. J. Geriatr. Psychiatry.

[23] Suzuki T., Shimada H., Makizako H., Doi T., Yoshida D., Ito K., Shimokata H., Washimi Y., Endo H., Kato T. (2013). A Randomized Controlled Trial of Multicomponent Exercise in Older Adults with Mild Cognitive Impairment. PLoS ONE.

[24] Nuzum H., Stickel A., Corona M., Zeller M., Melrose R.J., Wilkins S.S. (2020). Potential Benefits of Physical Activity in MCI and Dementia. Behav. Neurol..

[25] Sachdeva A., Kumar K., Anand K.S. (2015). Non Pharmacological Cognitive Enhancers—Current Perspectives. J. Clin. Diagn. Res..

[26] Gallardo-Gómez D., Del Pozo-Cruz J., Noetel M., Álvarez-Barbosa F., Alfonso-Rosa R.M., Del Pozo Cruz B. (2022). Optimal Dose and Type of Exercise to Improve Cognitive Function in Older Adults: A Systematic Review and Bayesian Model-Based Network Meta-Analysis of RCTs. Ageing Res. Rev..

[27] Kirk-Sanchez N.J., McGough E.L. (2014). Physical Exercise and Cognitive Performance in the Elderly: Current Perspectives. Clin. Interv. Aging.

[28] Klimova B., Dostalova R. (2020). The Impact of Physical Activities on Cognitive Performance among Healthy Older Individuals. Brain Sci..

[29] Biazus-Sehn L.F., Schuch F.B., Firth J., Stigger F.d.S. (2020). Effects of Physical Exercise on Cognitive Function of Older Adults with Mild Cognitive Impairment: A Systematic Review and Meta-Analysis. Arch. Gerontol. Geriatr..

[30] Deodato M., Granato A., Buoite Stella A., Martini M., Marchetti E., Lise I., Galmonte A., Murena L., Manganotti P. (2024). Efficacy of a Dual Task Protocol on Neurophysiological and Clinical Outcomes in Migraine: A Randomized Control Trial. Neurol. Sci..

[31] Deodato M., Qualizza C., Martini M., Mazzari L., Furlanis G., Buoite Stella A., Manganotti P. (2024). Efficacy of Dual-Task Augmented Reality Rehabilitation in Non-Hospitalized Adults with Self-Reported Long COVID Fatigue and Cognitive Impairment: A Pilot Study. Neurol. Sci..

[32] Veronese N., Soysal P., Demurtas J., Solmi M., Bruyère O., Christodoulou N., Ramalho R., Fusar-Poli P., Lappas A.S., Pinto D. (2023). Physical Activity and Exercise for the Prevention and Management of Mild Cognitive Impairment and Dementia: A Collaborative International Guideline. Eur. Geriatr. Med..

[33] Heilmann F., Weinberg H., Wollny R. (2022). The Impact of Practicing Open- vs. Closed-Skill Sports on Executive Functions-A Meta-Analytic and Systematic Review with a Focus on Characteristics of Sports. Brain Sci..

[34] Oishi A., Yamasaki T. (2024). Benefits of Badminton for Preventing Cognitive Decline and Dementia. Encyclopedia.

[35] Yamasaki T. (2023). Benefits of Judo Training for Brain Functions Related to Physical and Cognitive Performance in Older Adults. Encyclopedia.

[36] Yang J.-G., Thapa N., Park H.-J., Bae S., Park K.W., Park J.-H., Park H. (2022). Virtual Reality and Exercise Training Enhance Brain, Cognitive, and Physical Health in Older Adults with Mild Cognitive Impairment. Int. J. Environ. Environ. Res. Public Health.

[37] Park H.-J., Thapa N., Bae S., Yang J.-G., Choi J., Noh E.-S., Park H. (2024). Association between Physical Function, Mental Function and Frailty in Community-Dwelling Older Adults: A Cross-Sectional Study. J. Clin. Med..

[38] Di Lorito C., Bosco A., Booth V., Goldberg S., Harwood R.H., Van der Wardt V. (2020). Adherence to Exercise Interventions in Older People with Mild Cognitive Impairment and Dementia: A Systematic Review and Meta-Analysis. Prev. Med. Rep..

[39] O’Neil-Pirozzi T.M., Cattaneo G., Solana-Sánchez J., Gomes-Osman J., Pascual-Leone A. (2022). The Importance of Motivation to Older Adult Physical and Cognitive Exercise Program Development, Initiation, and Adherence. Front. Aging.

[40] Campbell M.J., Toth A.J., Moran A.P., Kowal M., Exton C. (2018). eSports: A New Window on Neurocognitive Expertise?. Prog. Brain Res..

[41] Lachowicz M., Żurek A., Jamro D., Serweta-Pawlik A., Żurek G. (2024). Changes in Concentration Performance and Alternating Attention after Short-Term Virtual Reality Training in E-Athletes: A Pilot Study. Sci. Rep..

[42] Sokołowska B. (2023). Impact of Virtual Reality Cognitive and Motor Exercises on Brain Health. Int. J. Environ. Res. Public Health.

[43] Hajebrahimi F., Velioglu H.A., Bayraktaroglu Z., Helvaci Yilmaz N., Hanoglu L. (2022). Clinical Evaluation and Resting State fMRI Analysis of Virtual Reality Based Training in Parkinson’s Disease through a Randomized Controlled Trial. Sci. Rep..

[44] Bae S., Shimada H., Lee S., Makino K., Chiba I., Katayama O., Harada K., Park H., Toba K. (2023). Subjective Cognitive Decline and Frailty Trajectories and Influencing Factors in Japanese Community-Dwelling Older Adults: A Longitudinal Study. J. Clin. Med..

[45] Sañudo B., Abdi E., Bernardo-Filho M., Taiar R. (2020). Aerobic Exercise with Superimposed Virtual Reality Improves Cognitive Flexibility and Selective Attention in Young Males. Appl. Sci..

[46] Toth A.J., Ramsbottom N., Kowal M., Campbell M.J. (2020). Converging Evidence Supporting the Cognitive Link between Exercise and Esport Performance: A Dual Systematic Review. Brain Sci..

[47] Thapa N., Yang J.-G., Bae S., Kim G.-M., Park H.-J., Park H. (2022). Effect of Electrical Muscle Stimulation and Resistance Exercise Intervention on Physical and Brain Function in Middle-Aged and Older Women. Int. J. Environ. Res. Public Health.

[48] Rosa P.J., Sousa C., Faustino B., Feiteira F., Oliveira J., Lopes P., Ganito P., Morais D. The Effect of Virtual Reality—Based Serious Games in Cognitive Interventions: A Meta—Analysis Study. Proceedings of the 4th Workshop on ICTs for Improving Patients Rehabilitation Research Techniques; Association for Computing Machinery.

[49] Thapa N., Park H.J., Yang J.-G., Son H., Jang M., Lee J., Kang S.W., Park K.W., Park H. (2020). The Effect of a Virtual Reality-Based Intervention Program on Cognition in Older Adults with Mild Cognitive Impairment: A Randomized Control Trial. J. Clin. Med..

[50] Stanmore E., Stubbs B., Vancampfort D., de Bruin E.D., Firth J. (2017). The Effect of Active Video Games on Cognitive Functioning in Clinical and Non-Clinical Populations: A Meta-Analysis of Randomized Controlled Trials. Neurosci. Biobehav. Rev..

[51] Ferreira S., Raimundo A., Pozo-Cruz J.D., Bernardino A., Leite N., Yoshida H.M., Marmeleira J. (2024). Effects of Multimodal Exercise WITH Augmented Reality on Cognition in Community-Dwelling Older Adults. J. Am. Med. Dir. Assoc..

[52] Sokolov A.A., Collignon A., Bieler-Aeschlimann M. (2020). Serious Video Games and Virtual Reality for Prevention and Neurorehabilitation of Cognitive Decline Because of Aging and Neurodegeneration. Curr. Opin. Neurol..

[53] Sakhare A., Stradford J., Ravichandran R., Deng R., Ruiz J., Subramanian K., Suh J., Pa J. (2021). Simultaneous Exercise and Cognitive Training in Virtual Reality Phase 2 Pilot Study: Impact on Brain Health and Cognition in Older Adults. Brain Plast..

[54] Richlan F., Weiß M., Kastner P., Braid J. (2023). Virtual Training, Real Effects: A Narrative Review on Sports Performance Enhancement through Interventions in Virtual Reality. Front. Psychol..

[55] Bediou B., Adams D.M., Mayer R.E., Tipton E., Green C.S., Bavelier D. (2018). Meta-Analysis of Action Video Game Impact on Perceptual, Attentional, and Cognitive Skills. Psychol. Bull..

[56] Green C.S., Bavelier D. (2012). Learning, Attentional Control, and Action Video Games. Curr. Biol..

[57] Kowal M., Toth A.J., Exton C., Campbell M.J. (2018). Different Cognitive Abilities Displayed by Action Video Gamers and Non-Gamers. Comput. Hum. Behav..

[58] Palaus M., Marron E.M., Viejo-Sobera R., Redolar-Ripoll D. (2017). Neural Basis of Video Gaming: A Systematic Review. Front. Hum. Neurosci..

[59] Sala G., Tatlidil K.S., Gobet F. (2018). Video Game Training Does Not Enhance Cognitive Ability: A Comprehensive Meta-Analytic Investigation. Psychol. Bull..

[60] Boot W.R., Kramer A.F., Simons D.J., Fabiani M., Gratton G. (2008). The Effects of Video Game Playing on Attention, Memory, and Executive Control. Acta Psychol..

[61] Voss M.W., Prakash R.S., Erickson K.I., Boot W.R., Basak C., Neider M.B., Simons D.J., Fabiani M., Gratton G., Kramer A.F. (2012). Effects of Training Strategies Implemented in a Complex Videogame on Functional Connectivity of Attentional Networks. Neuroimage.

[62] Mayer R.E. (2014). Computer Games for Learning: An Evidence-Based Approach.

[63] Stafford T., Dewar M. (2014). Tracing the Trajectory of Skill Learning with a Very Large Sample of Online Game Players. Psychol. Sci..

[64] Wang P., Liu H.-H., Zhu X.-T., Meng T., Li H.-J., Zuo X.-N. (2016). Action Video Game Training for Healthy Adults: A Meta-Analytic Study. Front. Psychol..

[65] Latham A.J., Patston L.L.M., Tippett L.J. (2013). The Virtual Brain: 30 Years of Video-Game Play and Cognitive Abilities. Front. Psychol..

[66] Gentile D.A., Choo H., Liau A., Sim T., Li D., Fung D., Khoo A. (2011). Pathological Video Game Use among Youths: A Two-Year Longitudinal Study. Pediatrics.

[67] King D.L., Gradisar M., Drummond A., Lovato N., Wessel J., Micic G., Douglas P., Delfabbro P. (2013). The Impact of Prolonged Violent Video-Gaming on Adolescent Sleep: An Experimental Study. J. Sleep. Res..

[68] Moisala M., Salmela V., Hietajärvi L., Salo E., Carlson S., Salonen O., Lonka K., Hakkarainen K., Salmela-Aro K., Alho K. (2016). Media Multitasking Is Associated with Distractibility and Increased Prefrontal Activity in Adolescents and Young Adults. Neuroimage.

[69] Takeuchi H., Taki Y., Hashizume H., Asano K., Asano M., Sassa Y., Yokota S., Kotozaki Y., Nouchi R., Kawashima R. (2016). Impact of Videogame Play on the Brain’s Microstructural Properties: Cross-Sectional and Longitudinal Analyses. Mol. Psychiatry.

[70] Weinstein A.M. (2017). An Update Overview on Brain Imaging Studies of Internet Gaming Disorder. Front. Psychiatry.

[71] DiFrancisco-Donoghue J., Balentine J., Schmidt G., Zwibel H. (2019). Managing the Health of the eSport Athlete: An Integrated Health Management Model. BMJ Open Sport. Exerc. Med..

[72] Liu L., Yao Y.-W., Li C.-S.R., Zhang J.-T., Xia C.-C., Lan J., Ma S.-S., Zhou N., Fang X.-Y. (2018). The Comorbidity Between Internet Gaming Disorder and Depression: Interrelationship and Neural Mechanisms. Front. Psychiatry.

[73] Kowert R., Domahidi E., Quandt T. (2014). The Relationship between Online Video Game Involvement and Gaming-Related Friendships among Emotionally Sensitive Individuals. Cyberpsychol. Behav. Soc. Netw..

[74] Przybylski A.K. (2014). Electronic Gaming and Psychosocial Adjustment. Pediatrics.

[75] Soldevila-Domenech N., Ayala-Garcia A., Barbera M., Lehtisalo J., Forcano L., Diaz-Ponce A., Zwan M., van der Flier W.M., Ngandu T., Kivipelto M. (2025). Adherence and Intensity in Multimodal Lifestyle-Based Interventions for Cognitive Decline Prevention: State-of-the-Art and Future Directions. Alzheimers Res. Ther..

